# Investigations Concerning the Quality of the Water Supply at Memphis and Other Places in Tennessee and Mississippi, under Direction of the National Board of Health

**Published:** 1880-04

**Authors:** Charles Smart

**Affiliations:** Captain and Assistant Surgeon, U. S. A.


### Notes and Extracts.

---

INVESTIGATIONS CONCERNING THE QUALITY OF THE WATER SUPPLY AT MEMPHIS AND OTHER PLACES IN TENNESSEE AND MISSISSIPPI, UNDER THE DIRECTION OF THE NATIONAL BOARD OF HEALTH.

Having long been satisfied that our ability to prevent or limit the progress of epidemic as well as endemic diseases, will depend mostly upon the extent and accuracy of our knowledge concerning the local sanitary conditions existing where such diseases prevail, we were especially gratified to find that among the several important lines of inquiry, instituted by the National Board of Health, was one for examining the quality of the water supply in many of the places in the lower Mississippi Valley, in which yellow fever prevailed in the summers of 1878 and '79. The work has been prosecuted with considerable zeal, under the direction of Dr. Smart, whose report on the subject we give below, copied from "Supplement No. 3," of National Board of Health Bulletin.

While the investigation is by no means complete, and the report gives only sanitary results, instead of detailed analyses, it is quite sufficient to show that both the soil and the water supply of Memphis and many other places named in the report, are largely impregnated with organic matter highly pernicious to the public health. It is not at all difficult to understand why a population using such water, and breathing the emanations from a soil so impregnated, should be subject to severe epidemics in seasons of high temperature, and to a high ratio of mortality at all times. We say a high ratio of mortality at all times, because it is a great mistake to suppose that a soil impregnated with sewage matter and water contaminated, exert an influence only on special epidemics. On the contrary, such circumstances occasion a greater aggregate amount of sickness and mortality by increasing the number and fatality of attacks of ordinary diseases than by special epidemics. For instance, in turning directly to the table showing the weekly mortality of different cities in the United States, that for the week ending February 28th, for instance, we find the number of deaths for each 1,000 of the population, to be, in Boston, 23.1; Providence, 21.4; New York, 25.7; Philadelphia, 17.0; Baltimore, 14.4; Cincinnati, 17.3; Chicago, 16.4; Louisville, 12.3; Memphis, 37.3.

The following is the report, which will not only repay for the time of perusal, but will be useful for reference hereafter:

### REPORT OF DR. CHARLES SMART.

WASHINGTON, D. C., Dec. 30, 1879.

SIR: I have the honor to submit the following report of the practical results of my analysis of the water supplies of certain towns and villages in Mississippi and Tennessee.

My instructions did not require an exhaustive chemical analysis of the various waters with the quantitative determination of the saline matters which might be present in them. There was contemplated only what has been called a *sanitary analysis*, an investigation into the question of wholesomeness or unwholesomeness. But as a water which is unfit for potable uses from excess of metallic, earthy, or alkaline salts can usually be detected by its taste or hardness, a sanitary examination becomes practically an investigation into the organic matter which is contained in the sample.

The presence of organic substances can easily be detected in most waters, for there are few which are organically pure; but there is no royal road to the estimation of the quality, nor to—what is of as much importance—an appreciation of the quality. The examination must consist in instituting a series of experiments on the organic matter, on the substances which accompany it in the water, and on those derived from it. These various witnesses are, as it were, interrogated, and from a consideration of their testimony an opinion is formed as to the quantity of the organic contamination, as to its origin in the animal or vegetable kingdom, as to its source, whether near or remote—in a word, as to the wholesomeness or unwholesomeness of the water which contains it.

The examination being of necessity complex, and each point susceptible of determination by different processes, all of which have their advocates, a consultation was held with Prof. Ira Remsen and Dr. Harmon K. Morse, of the Johns Hopkins University, concerning the ground to be covered by the examination in each instance and the best mode of accomplishing it, consistent with portability of apparatus and other considerations. I am especially indebted to these gentlemen for calling my attention to Tiemann's modification of Schulze's process for the estimation of nitrates, which they have used for some time in their water examinations, and the accuracy of which they have practically determined.

The experimental points decided upon as essential were—

*First.* The total solids as obtained by the balance after the evaporation of a given quantity of water;

*Second.* The separation of this total by ignition and the balance into substances dissipated by heat, such as organic matter, nitrates, etc., and the inorganic residue;

*Third.* An estimation of the amount of oxygen required to oxidize the oxidizable substances present in the water, as affording a view of the organic matter from one side; while a view of the same impurity was to be obtained on another aspect by—

*Fourth.* The break-up of the organic substances and estimation of their nitrogen in the form of albuminoid ammonia;

*Fifth.* The quantity of free ammonia present;

*Sixth.* That of nitrous acid;

*Seventh.* Of nitric acid;

*Eight.* Of chlorine; and,

*Lastly.* The microscopic appearance of the sediment.*

With these data and a knowledge of what might be termed the natural history of the water, whether rain, well, spring, river

* Statistics of morbility and mortality, as gathered by the house-to-house inspection, were so largely matters of memory and not of record that they are not thought worth presenting in this connection.

water, etc., and of its surroundings, it was conceived that an accurate opinion could be given as to its quality, not by the operator only, but by any one conversant with the processes of water analysis, no matter what the method he adopted in the formation of his opinion.

In accordance with instructions, I proceeded to the town of Jackson, Miss., which I found mainly supplied by rain-water collected and stored in underground cisterns. As the time at my disposal did not admit of the examination of a sufficient number of these waters to enable an opinion to be given in general terms upon the character of the supply, I approximated to a generalization by selecting—

*First.* Water supplying public institutions, such as State penitentiary, the deaf and dumb asylum, the institute for the blind, etc.

*Second.* Public or semi-public cisterns.

*Third.* A few samples from the cisterns of well-to-do citizens.

*Fourth.* A few from those of the colored people.

*Fifth.* Samples from houses in which there was or had recently been malarial remittent fevers.

Thirteen samples were furnished and analyzed, with the following results:

| | Cisterns. | Water good. | Water fair. | Water bad. | Sewerage |
|---|---|---|---|---|---|
| Sound | 2 | ........ | 1 | 1 | ...... |
| Spring | 3 | 1 | 1 | 1 | ....... |
| Leaky | 8 | 4 | 1 | 3 | ....... |
| Total | 13 | 5 | 3 | 5 | ....... |

This is manifestly a bad showing—only two sound cisterns out of thirteen, one containing water rank with vegetable impurity, while the contents of the other are not above suspicion. That the cisterns are not contaminated with sewage matter is owing to purity of soil, and not to their own merits. Several of them are neither more nor less than shallow wells, receiving more of their contents by percolation than by inflow from above.

One well-water, from northwest Jackson, was received for examination. It was organically impure, even had its use not been contra-indicated by the amount of its earthy constituents.

Having been informed by Dr. Wirt Johnston, secretary of the State board of health, that during the 1878 epidemic of yellow fever the disease passed from Jackson eastward along the line of railroad and ravaged all the settlements along the track to Meridian, except the village of Brandon, I proceeded to that place and secured samples from the sources of supply most generally used. These consisted of three wells, two springs and one cistern. The last was sound, but contained bad water. The springs were good, but one (Youst's) would have been better had the arrangements for preserving it from surface admixture been other than of the most primitive character. Of the wells one furnished good, another fair or usable, and the third an impure water.

The next place at which my instructions called for an investigation was Grenada, Miss. Here the supply was found to be from wells averaging about twenty-five feet in depth. They were all free from excess of any earthy salts.

Eighteen samples were examined, of which fourteen were good waters, one fair and three bad. Of the last, one (Doak's) would probably have shown as a good water had a fair specimen been furnished for analysis. On investigating the well, after examination of the water, it was found that a new pump had been inserted on the day before the sample was collected, and that the well was unusually turbid from this interference. The two remaining on the record as bad waters were undoubtedly contaminated with sewage.

The comparative freedom of the wells in this town from sewage infiltration is owing to the absence of privy vaults. The Mississippi State Board requires the use of dry earth and surface receptacles, and Grenada has been very thorough in her adoption of this surface system.

For samples of the supply of small country settlements I proceeded from Grenada to Duckhill, Miss., where I examined three wells, the waters of which were good, although possessed at times of a sulphur taste on account of a blue-clay stratum which lies below the water-mark.

Returning from this place to Grenada, I collected at Elliott, Miss., samples from three wells, two of which were good and one bad.

At Payne's place, which is finely situated from a sanitary point of view, but which furnished twenty-five cases and thirteen deaths out of one hundred people living there during the 1878 epidemic, I obtained samples from two cisterns and one well. The latter furnished excellent water, but both cisterns, although sound, contained very impure supplies.

At Green's Chapel, the only well yielded a very satisfactory water.

If the above samples from country places can be admitted as illustrations of the well-water supply of such settlements generally, their freedom from organic impurity, from surface washings and sewage, is a matter for congratulation.

Grenada and its vicinity is thus seen to have an excellent water supply, the impurity in the few bad cases being due to local and preventable causes.

At Holly Springs, Miss., where examinations were next required to be instituted, twenty-five waters were analyzed, of which eighteen were from wells, five from underground cisterns, and two from springs.

The following tabulates the results:

| | Cisterns, etc. | Water good. | Water fair. | Water bad. | Sewage. |
|---|---|---|---|---|---|
| Sound | 3 | 3 | ........ | ........ | ....... |
| Siping | 1 | 1 | ........ | ........ | ....... |
| Leaky | 1 | ........ | ........ | 1 | ....... |
| Wells | 18 | 9 | 2 | 3 | 4 |
| Springs | 2 | 1 | ........ | 1 | ....... |
| Total | 25 | 14 | 2 | 5 | 4 |

Of the wells contaminated by sewage in this town, some are owing to pollution of soil by privy vaults now disused; one arises from the neighborhood of a vault which is yet in use, another from the immediate proximity of a cow-stable, combined with a break in the curb which admits of surface inflow in wet weather.

Some of the bad waters can have the organic matter contained in them accounted for only by the presence of large trees, the roots of which may lead surface water into the well with insufficient filtration, or, being in a state of decay, may charge the inflowing water with their detritus. In several of these cases the surface around the well and adjacent tree-trunks is garden ground freely manured with farm-yard refuse.

In passing from Mississippi, where privy vaults have been abolished in favor of the surface system, to Tennessee, where they continue in general use, the character of the well-water supply was found to undergo a marked change. Sewage pollution, which up to this time had been met with only in exceptional cases, was in Brownsville, Tennessee, discovered to be the general condition.

Eleven wells were examined, of which only two were good. Two furnished fair or usable water, one was bad from vegetable impurity, and six were found largely contaminated by sewage from vaults. Incidentally, it was noted by Dr. W. W. Taylor, secretary of the local board, that three cases of typhoid had occurred in a house (that of Judge Bond) supplied by one of these polluted wells.

Only two cisterns were examined, one of which was sound and contained a good water, while the other was leaky—a veritable shallow well—and contained a most impure supply.

In a thriving town like Brownsville, where the soil has become so contaminated as to infect the wells in this manner, it is high time for the establishment of a sewerage system. The vaults should be abolished in the mean time and the Mississippi surface system instituted. But this is beyond the limits of my inquiry, and is mentioned only in passing.

So far as the water supply is concerned, it is is imperative that some action be taken by the local board. The town is ripe for decimation by typhoid fever and other diseases which originate in impurity of soil and are propagated by impure water. The proper remedy is the organization of a water company and the introduction of a supply from a suitable source entirely beyond the risk of pollution by the consumers. But if this requires an expenditure exceeding the means of the town, recourse must be had in the mean time to a rain-water supply, or, in certain localities outside of the denser areas, to deep wells lined with brick to exclude infiltration from the surface.

The city of Memphis, Tennessee, was found to depend for its supply upon three unequal sources, which, in the order of their importance, were—

*First.* The underground cisterns, of which it was estimated that there were no less than four thousand in use within the city limits.

*Second.* The wells, of which there were quite a number, but many of them were not used on account of a bad repute, based principally on the hardness of the water, or the taste of its organic salts.

*Third.* The hydrant water, or that furnished from the Wolf river by the Memphis Water Company.

Eighty samples of cistern water, from various parts of the city, were examined, with the following result:

| | Cisterns. | Water good. | Water fair. | Water bad. | Sewage. |
|---|---|---|---|---|---|
| Sound | 36 | 25 | 8 | 3 | ...... |
| Siping | 12 | 2 | 1 | 2 | 7 |
| Leaky | 32 | 6 | 2 | ...... | 23 |
| Total | 80 | 33 | 11 | 5 | 31 |

But these cannot be viewed as illustrating the condition of the four thousand cisterns in the city, inasmuch as, although they came from all quarters, they were in every instance selected samples. Many were brought for examination by citizens whose very anxiety concerning their water supply might be looked upon as an argument in favor of the probability of the cistern being sound and the water pure. Other samples, as those from the public schools, were sent in mainly for the purpose of verifying their purity. On the other hand, samples brought by the medical inspectors for analysis were presented on account of their probable impurity, while the last nine of the waters which were examined were selected by the analyst from a list of those known by preliminary examination to be impure.

But while one may not generalize concerning the Memphis cisterns from this table, it shows conclusively that the impurity of the soil is such that when a leak exists in the cistern, the probabilities are strongly in favor of a pollution by sewage of the contained water.

As the complete sanitary analysis of a water occupied so much time, and as the number of cisterns was relatively so immense, a ready method was sought by which the condition of a cistern and the probable quality of its contents might be determined.

At first sight, the amount of total solids in a stored rain-water would seem to afford the means of judging as to the soundness of its cistern, any sipage or leakage of necessity carrying into the water earthy and other salts which would increase the total. But several instances occurred in the above analyses where lime carbonate in the residue came manifestly from the cement lining of a sound cistern. This method had therefore to be excluded as fallacious.

No objection, however, attached to an estimation of the chlorine present in the water as an index of the condition of the cistern. Rain-water contains a small proportion of chlorine, the amount varying with the condition of the atmosphere and the purity of the shedding surface. In the inland city of Memphis the amount naturally existing in its rainfall is not large. Rain which was shed from the roof of the Peabody Hotel, during a heavy fall on December 3, contained .075 parts of chlorine in the 100. A cistern water which does not contain more than this must be undeniably free from soil pollution, for the finished analysis showed the soil to be so charged with chlorides that the slightest siping or leakage was marked by an increased chlorine figure. A small excess over the normal might exist and the cistern be sound, the increase being due to an unusual foulness of the roof. As much as .15 parts of chlorine was shown to be consistent with soundness, but as the amount increased beyond this figure the probability of leakage became proportionally great. A series of chlorine determinations was then made on cistern samples collected from all the wards in the city; and, as no selection was exercised, the results may be viewed as expressing the condition of the Memphis cistern supply.

In tabulating them, those waters which contained less than

.075 parts of chlorine are set down as from *undoubtedly sound* cisterns, the supply itself being in all probability of good quality. In cases where the amount lay between .075 and .15 parts the cisterns are recorded as *probably sound*, the increase being due to vegetable contamination and foulness of the shedding surfaces, although in a small proportion of the cases it might be owing to a slight siping from the soil. Here the water must be considered of doubtful quality. In those instances where the chlorine figure lay between .15 and .30 parts, the cistern is reported as probably *siping*, the increase coming from the soil, and being in all likelihood a sewage accompaniment, although in rare cases it might arise from a large organic impurity without leakage on the part of the cistern. In either case the water is probably bad. Lastly, where the amount exceeded .30 parts in the 100 of water, the cistern is viewed as undoubtedly *leaky*, and as containing an impure water supply.

CISTERNS EXAMINED.

| | |
|---|---|
| Undoubtedly sound | 127 |
| Probably sound | 82 |
| Probably siping | 82 |
| Undoubtedly leaky | 158 |
| Total | 449 |

In estimating the condition of the Memphis water supply from this source, there should be added to the above figures nine leaky cisterns, making 167 in a total of 458. These nine samples were brought in for the chlorine experiment along with the others, but a complete sanitary analysis having been subsequently made to determine the characters of their impurity, they were removed from the list of cisterns examined to that of cistern-waters analyzed.

The large proportion of leaky cisterns, with the strong probability of sewage contamination in each instance, requires that some action be taken to insure a better water supply for the city. But whatever may ultimately be done in this direction, the citizens will have to depend upon their cisterns for some time to come. Yet the character of many of these waters is such as to call, in the interest of the public health, for an immediate interference. In several instances I was enabled, by personal communication with the owners, to warn and advise. But in other cases, where the water was brought by the inspecting officers, I did not have the means of notifying the consumers. To overcome this difficulty and enable action to be taken in the cases of certain cisterns, which from their excessive leakage appeared to require immediate attention, I sent a communication embracing some of the facts here recorded to Dr. R. W. Mitchell, member of the National Board of Health, and of the committee on the sanitary survey of Memphis, with a list of sixty-five of the worst cisterns and a map giving the location and character of the whole number examined.

But the facts developed by the analysis constitute only a small part of the total array which demands investigation in the sanitary interests of the city. Among the thirty-five hundred unexamined cisterns there at least twelve hundred which leak. These should be singled out by the local authorities by means of the chlorine test, and their abandonment should be ordered if there is manifest danger from soil pollution in their surroundings; but if there is no such danger it would be sufficient to direct them to be cleaned out and re-lined with a thick coating of Portland cement.

The analyses of the well-waters demonstrated the impurity of the city's soil in as marked a manner as did those of the leaking cisterns. Of nineteen wells, all of which were reported as brick-lined to exclude infiltration, fourteen contained sewage matter, either in the recent state or oxidized by its passage through the soil. Of the five remaining, the use of one was contra-indicated by an excess of earthy salts, so that there were in reality only four waters, out of this list of nineteen, which could be warranted as fit for use. Of those which showed the presence of organic mat- in an oxidized condition, one—Pontotoc—was so free from recent contamination, and at the same time contained such a small proportion of oxidized matter, that the *water* might be considered as wholesome. The *well*, however, must be looked upon as dangerous, inasmuch as an increased flow of water into it might at any time bring unaltered sewage to pollute the supply.

The waters might be thus classified :

Four good in every respect.

One doubtful, as liable at any time to contamination.

Eight bad in every respect.

Four potable so far as recent organic matter is concerned, but condemned on account of oxidized organic matter and excess of inorganic salts.

One unwholesome solely on account of dissolved inorganic salts.

One potable so far as inorganic constituents are concerned, but condemned on account of organic pollution.

In connection with the cistern and well-water supply of Memphis I have especially to thank Dr. R. W. Mitchell, member of the National Board of Health, and Dr. Frank W. Reilly, superintendent of the house-to-house inspection, for the assistance they furnished, in securing the necessary samples for analysis, and for many valuable suggestions connected with my work.

Besides the cisterns and wells above enumerated, the examinations at Memphis included one water from a tank or reservoir built above ground, one rain-water collected direct from a roof, in order to place on the record the sanitary analysis of the rainfall which was then filling the cisterns, and thirteen samples of river water, one from Elmwood Creek, three from the Mississippi, and nine from the Wolf river.

The supply furnished by the Memphis Water Company is drawn from Wolf river, a short distance above its mouth. It is pumped from the river and distributed without any intermediate process of purification.

During the period of my stay in Memphis, Wolf River was an uninviting stream, turbid with particles of red clay, which, on account of their extreme minuteness, rendered filtration difficult from the tendency of the particles to clog the filter—and sedimentation practically impossible. Entangled with the clay there was much vegetable matter, which could be separated only by the separation of the mineral particles. Hence, analyses of the water made on samples taken on November 12th and 18th and December 2nd and 15th showed it to be unwholesome in a high degree. Many householders who were doubtful as to the condition of their cisterns made use of this water after passing it through a charcoal filter. One such filtered sample was examined and found to be a specimen of very pure water. Those citizens only who made use of the filtered water had an interest in this result, which is to be set to the credit of the charcoal, not to that of the water. Sedimentation was accomplished on the small scale in a few instances by filling cisterns with the river water at a time when the stream was low and comparatively clear. Three samples of water stored in this manner were examined and found to be of good quality. But the long-continued sedimentation in a clean cistern which is to be credited with this result is a process which, like the charcoal filtration, can be accomplished only in individual cases.

It is, no doubt, true that at other seasons of the year this stream is less impure than at present, but a water, to be suitable for the supply of a large city, should be pure at all seasons, or, if not, it should at least be susceptible of purification on the large scale. The Wolf River water, unfortunately, cannot be thus purified. Any filter which might be constructed would be clogged within a few hours, and a series of reservoirs, to permit of subsidence, would require to be of extravagant size unless some means were devised to hasten the process.

With the view of ascertaining the character of the water higher up the stream than the location of the water-works, a sample was procured on November 18th from Raleigh, a point nine miles distant from the city. This specimen was found to be somewhat less impure than that collected on the same day near the water company's inflow. But it was unwholesome, from an excess of vegetable matter, and as objectionable as the other when viewed as a possible source of supply. It was loaded with the same red clay which renders the hydrant-water so difficult of purification.

In order to effect a comparison between the waters of the Mississippi and Wolf Rivers, Capt. W. H. H. Benyaurd, Corps of Engineers, United States Army, who furnished the samples already mentioned, collected specimens of the Mississippi on November 18th and December 2nd and 15th. But these were insufficient for the purpose. The periods of flood in the two streams are not coincident. On the first of the above dates Wolf River was somewhat swollen, while the rise in the Mississippi had not commenced. The Mississippi water was then a fair sample of river water, containing a certain amount of vegetable impurity, but by no means so much as has been recorded as existing in some Western streams of good repute as to wholesomeness. On the second date the Mississippi sample, although very turbid, the river being 7½ feet above low water mark, and rising—was comparatively free from organic matter. On the third occasion, the river had risen to 10 feet and was exceedingly turbid. When the Mississippi is at this height, its waters impede the outflow from Wolf River, causing stagnation and flooding in the mouth of the latter. The sample presented the same amount of organic impurity as that furnished by the Wolf River water collected on the same day, enough to condemn it as unfit for potable use.

To establish a comparison between the streams, the annual lines of organic impurity should be determined. But continued observations of this nature do not appear to be called for to decide the question as to which is the better water for a town supply. Both are pure when at low-water mark, and free from impurity. Both are impure when swollen and turbid. So far they agree. But the red clay of the smaller stream rises into turbidity more readily and falls more slowly than the more siliceous sedimentary matters of the Mississippi. This would suggest that the annual period of turbidity and consequent impurity is less in the Mississippi. But the possibility of purifying a supply drawn from it should cause it to be preferred, even if its period of turbidity were greater than that of Wolf River. So far as dissolved inorganic substances are concerned, both waters are of good quality.

This report has, of course, no consideration for the engineering and financial sides of the question.

I have the honor to accompany this report with an itemized list of the various well, spring, river and cistern waters analyzed, and of the Memphis cisterns tested chemically for leakage. I also append a copy of the letter referred to above as having been sent to Dr. R. W. Mitchell with the view of reaching the local authorities of Memphis.

I have the honor to be, very respectfully, your obedient servant,

CHARLES SMART,

*Captain and Assistant Surgeon, U. S. A.*

